# In utero hematopoietic cell transplantation for hemoglobinopathies

**DOI:** 10.3389/fphar.2014.00278

**Published:** 2015-01-12

**Authors:** S. Christopher Derderian, Cerine Jeanty, Mark C. Walters, Elliott Vichinsky, Tippi C. MacKenzie

**Affiliations:** ^1^Department of Surgery, Eli and Edythe Broad Center of Regeneration Medicine, University of California San FranciscoSan Francisco, CA, USA; ^2^Children’s Hospital and Research Center OaklandOakland, CA, USA

**Keywords:** in utero transplantation, fetal therapy, alpha thalassemia, chimerism, tolerance

## Abstract

In utero hematopoietic cell transplantation (IUHCTx) is a promising strategy to circumvent the challenges of postnatal hematopoietic stem cell (HSC) transplantation. The goal of IUHCTx is to introduce donor cells into a naïve host prior to immune maturation, thereby inducing donor–specific tolerance. Thus, this technique has the potential of avoiding host myeloablative conditioning with cytotoxic agents. Over the past two decades, several attempts at IUHCTx have been made to cure numerous underlying congenital anomalies with limited success. In this review, we will briefly review the history of IUHCTx and give a perspective on alpha thalassemia major, one target disease for its clinical application.

## HISTORY OF IUHCTx

In utero hematopoietic cell transplantation offers the benefit of treating congenital stem cell disorders prior to birth while avoiding host myeloablative conditioning with cytotoxic agents (Golombeck et al., 2006; Vrecenak et al., 2014). The idea that exposure to foreign antigens can lead to tolerance was first recognized by Owen (1945), with the discovery that monochorionic dizygotic cattle were tolerant of long-lived chimeric cells from their siblings. Since then, natural chimerism has been described in both human and non-human primates (Picus et al., 1985; van Dijk et al., 1996), although it was not until the late 1970s that Fleischman and Mintz reported the first successful chimerism resulting from IUHCTx. Using a c-Kit deficient mouse which resulted in genetic anemia, they were able to reverse the anemia by transplanting adult allogeneic bone marrow stem cells into the placenta (Fleischman and Mintz, 1979). Since then, IUHCTx has proven to be successful in many animal models including mice (Carrier et al., 1995), goats (Pearce et al., 1989), dogs (Blakemore et al., 2004; Peranteau et al., 2009; Vrecenak et al., 2014), sheep (Flake et al., 1986), and non-human primates (Harrison et al., 1989; Tarantal et al., 2000). Mouse models have been used to manipulate various aspects of the maternal (Merianos et al., 2009; Nijagal et al., 2011) and fetal (Misra et al., 2009; Nijagal et al., 2013) immune systems to understand the mechanism of tolerance induction. In the large animal models, which are a necessary step to understanding the effects of immune ontogeny of human fetuses, high dose transplantation has enabled achieving clinically relevant levels of chimerism (Vrecenak et al., 2014).

In humans, the first successful IUHCTx was performed for bare lymphocyte syndrome (Touraine et al., 1989). Successful transplantation of fetuses with severe combined immunodeficiency (SCID) was also achieved by several groups (Flake et al., 1996; Wengler et al., 1996). However, subsequent attempts into fetuses with various disease processes including hemoglobinopathies, chronic granulomatous disease, Chediak–Higashi syndrome and inborn errors of metabolism were met with limited success (reviewed in Vrecenak and Flake, 2013). These limitation have led several groups to explore barriers to engraftment which include the fetal and maternal immune systems, the competitive disadvantage of donor cells when transplanted into an intact fetal host, and a lack of space within hematopoietic niches (reviewed in Nijagal et al., 2012). Since it has been shown that the maternal immune system (both T cells and B cells) is a critical barrier to engraftment (Merianos et al., 2009; Nijagal et al., 2011), clinical efforts should focus on transplantation of maternal (or maternally matched) hematopoietic cells. The levels of engraftment can also be increased by transplanting a high number of CD34 enriched, CD3 depleted bone marrow cells using an intravascular (as opposed to intraperitoneal) approach (Vrecenak et al., 2014). Further efforts to improve the competitive advantage of the transplanted cells and to create space for their engraftment in the hematopoietic niche will likely be necessary. For example, we have recently demonstrated that selective in utero depletion of host HSCs using an antibody against the c-Kit receptor (ACK2) results in therapeutic levels of engraftment after neonatal transplantation (Derderian et al., 2014), providing a proof of concept for such a conditioning approach in the fetal environment. This approach may also avoid the need for conventional myeloablative drugs such as busulfan that could cause tissue cytotoxicity in utero. Finally, transplantation prior to the development of circulating T cells is likely critical and further measures to promote fetal tolerance induction for example, by co-transplantation of regulatory T cells, should be explored.

## THERAPEUTIC POTENTIAL OF IUHCTx FOR ALPHA-THALASSEMIA

In utero hematopoietic cell transplantation has excellent potential to treat common hemoglobinopathies such as sickle cell disease and thalassemias. In particular, alpha thalassemia major can be diagnosed early in gestation and poses risks to the developing fetus including hydrops fetalis, which may provide further justification for an in utero intervention.

Alpha-thalassemia is one of the most common single-gene disorders, affecting approximately 5% of people worldwide (Lau et al., 1997; Chui and Waye, 1998; Leung et al., 2008). It is an autosomal recessive disease, resulting from DNA sequence deletions on chromosome 16. At least 40 deletions are known (reviewed in Vichinsky, 2009), the most common of which is the Southeast Asian deletion (-^SEA^; Chui and Waye, 1998; Hoppe, 2009). Since there are 4 alleles coding for the alpha-globin protein, the disease can present as a spectrum. The homozygous form (-/-), often referred to as Hb Bart’s, results in the absence of all alpha-globin production. Unaffected chains accumulate and form tetramers unable to transport oxygen, ultimately leading to hypoxia, non-immune fetal hydrops, and in utero demise (Leung et al., 2008).

### EARLY DIAGNOSIS IN UTERO

Advancements in prenatal diagnostic tools have provided means for early diagnosis of many congenital anomalies, including alpha-thalassemia. Anemia caused by alpha-thalassemia can be detected on ultrasound by an increase in the cardiothoracic ratio, an increase in middle cerebral artery peak systolic velocities, and the presence of non-immune hydrops. These changes have been detected as early as 12 weeks’ gestation (Lam et al., 1999; Li et al., 2007), which is well within the window of optimal timing for IUHCTx. Once anemia is suggested on ultrasound, the diagnosis of alpha-thalassemia requires fetal DNA for genetic sequencing. Currently, the most common modalities to obtain fetal DNA for analysis are amniocentesis, which can be performed as early as 16 weeks’ gestation with only a 0.5% risk of fetal demise (No authors, 1976), or chorionic villus sampling, which is performed as early as 10 weeks’ gestation (Nicolaides et al., 1994; Sundberg et al., 1997). More recently, genetic disorders have been diagnosed using cell-free fetal DNA, which is detectable in maternal serum as early as 7 weeks’ gestation (Lo et al., 1998). Advances in laboratory technology have increased the likelihood that we will soon be able to reliably diagnose alpha thalassemia major prenatally with maternal plasma (Sirichotiyakul et al., 2012; Ge et al., 2013). Although this strategy has great potential, detecting complex mutations of alpha-thalassemia major remains a challenge. It appears likely soon women at risk for carrying a fetus with Hb Bart’s will have the opportunity to undergo cell-free fetal DNA testing not only before the onset of fetal hydrops but at a time when the fetus is still in an immune tolerant state (Ge et al., 2013).

### IN UTERO MANIFESTATION

Fetuses with Hb Bart’s produce aberrant alpha-globin, which results in accumulation of dysfunctional hemoglobin tetramers, and impaired oxygen transportation. Definitive erythrocytes, composed predominantly of fetal hemoglobin (α_2_γ_2_), begin circulating at 10 weeks’ gestation (Migliaccio and Papayannopoulou, 2001). In utero, Hb Bart’s leads to anemia, heart failure, fetal growth restriction, oligohydramnios, and non-immune hydrops (Fucharoen et al., 1991), which historically was considered to be a harbinger of fetal demise (Laros, 1994). More recently, in utero exchange transfusion, which removes the dysfunctional hemoglobin, has been shown to reverse anemia, fetal growth restriction, and oligohydramnios (Dwinnell et al., 2011). However, this temporizing therapy is directed toward symptom relief and not curing the underlying disorder. An alternative strategy would be to offer IUHCTx to cure the genetic anemia even before the onset of any symptoms.

### CURRENT IN UTERO THERAPY

Nearly 20 documented cases of Hb Bart’s have been treated with in utero transfusion and outcomes have been generally favorable (Carr et al., 1995; Singer et al., 2000; Zhou et al., 2001; Lucke et al., 2005; Weisz et al., 2009; Yi et al., 2009; Dwinnell et al., 2011). However, these children are transfusion dependent and require iron chelators to prevent complications resulting from iron overload such as cirrhosis and insulin dependent diabetes. Neonatal complications include cognitive and limb reduction defects (Dwinnell et al., 2011). Among fetuses who do not undergo blood transfusions and survive to birth, 25–50% are affected by neurological or developmental shortcomings (Lucke et al., 2005; Lee et al., 2007), presumably from prolonged in utero hypoxemia. However, fetuses transfused early in gestation have a much lower incidence of cognitive and limb reduction defects. Despite our awareness that this process begins in utero, the only prenatal therapy available is in utero transfusions, which is merely directed at symptom relief.

## CLINICAL EXPERIENCE WITH IUHCTx FOR ALPHA-THALASSEMIA

There have been three attempts to treat alpha-thalassemia with IUHCTx (**Table 1**) and only one has demonstrated donor cell chimerism on autopsy. Each case used various strategies, making them difficult to compare. The timing of in utero transplantation differed, with cases #1 and #3 performed earlier in gestation (13 and 15 weeks, respectively) while case #2 was performed later (18 weeks). The source of donor cells differed as well. Case #2 used maternally derived bone marrow HSCs and was the only one with evidence of microchimerism on autopsy (termination was pursued at 24 weeks’ gestation after no evidence of engraftment was demonstrated by cord blood sampling). This observation is supported by experiments in mice demonstrating that maternally derived HSCs engraft better than paternally derived HSCs (Merianos et al., 2009; Nijagal et al., 2011).

**Table 1:** In utero transplantation for alpha-thalassemia.

Case	GA at diagnosis (weeks)	GA at transplant (weeks)	Cell source	Cell number	Route	Engraftment	Reference
1	10	13, 19, and 24	Paternal CD34^+^ BM cells	3 × 10^6^/kg	i.p., i.v., i.v.	CB at Birth and BM at 3 months – detectable alpha globin but no donor cell engraftment detected	Hayward et al. (1998)
2	N/A	18	Maternal *T*-cell depleted BM	6.3 × 10^8^	i.p.	CB at 20, 22, and 24 weeks – no engraftment Extramedullary engraftment on autopsy	Cowan and Golbus (1994)
3	13	15, 31	Cryopreserved FL from abortions between 5 and 10 weeks’ gestation	2.2 × 10^10,^ 2.7 × 10^10^	i.p., i.v.	CB at 29 weeks GA – no donor DNA or alpha globin No postnatal donor cell engraftment detected	Westgren et al. (1996)

*GA, gestational age; BM, bone marrow; kg, kilogram; i.p., intraperitoneal; i.v., intravenous; CB, cord blood; FL, fetal liver. N/A, not available*.

While we cannot draw any definitive conclusions from these attempts, strategies to improve engraftment are necessary. In each case, the first series of transplanted cells were injected into the peritoneal cavity, whereas evidence in animal models now supports that intravascular infusion is more likely to establish stable donor engraftment. Since fetuses with Hb Bart’s will be transfusion dependent, transplantation may be performed at the same time as an intrauterine transfusion. Based on animal models, transplantation of T-cell depleted, CD34 enriched maternal-derived HSCs should avoid a maternal immune response against the graft as well as taking advantage of pre-existing fetal tolerance to maternal cells. Additional areas to explore to improve engraftment are *ex vivo* manipulation to increase HSCs proliferative ability and homing potential [reviewed in Peranteau et al. (2009) in this issue] as well as fetal conditioning with non-myeloablative agents such as antibodies against the c-Kit receptor.

In summary, IUHCTx has only been successful in fetuses with SCID and the subsequent lack of success in other diseases has left the field undervalued. With advancements in technical strategies and a new repertoire of therapies, it is time to revisit the idea of IUHCTx for hemoglobinopathies. As with all fetal treatment endeavors, careful patient selection, meticulous attention to technical details, and accurate reporting of results will be critical to the success of future clinical trials.

## Conflict of Interest Statement

The authors declare that the research was conducted in the absence of any commercial or financial relationships that could be construed as a potential conflict of interest.
